# *MLL*-Rearranged Acute Leukemia with t(4;11)(q21;q23)—Current Treatment Options. Is There a Role for CAR-T Cell Therapy?

**DOI:** 10.3390/cells8111341

**Published:** 2019-10-29

**Authors:** Oliver Britten, Denise Ragusa, Sabrina Tosi, Yasser Mostafa Kamel

**Affiliations:** 1 Division of Biosciences, College of Health and Life Sciences, Institute of Environment, Health and Societies, Brunel University London, Uxbridge UB8 3PH, UK; 1630786@brunel.ac.uk (O.B.); denise.ragusa2@brunel.ac.uk (D.R.); 2 ASYS Pharmaceutical Consultants-APC Inc. 2, Bedford, Nova Scotia B4A 4L2, Canada; yasser@asyspc.com

**Keywords:** *MLL*, *mixed-lineage leukemia*, *KMT2A*, CAR-T cell therapy, acute leukemia, chromosome translocation, MLL-AF4

## Abstract

The *MLL* (*mixed-lineage leukemia*) gene, located on chromosome 11q23, is involved in chromosomal translocations in a subtype of acute leukemia, which represents approximately 10% of acute lymphoblastic leukemia and 2.8% of acute myeloid leukemia cases. These translocations form fusions with various genes, of which more than 80 partner genes for *MLL* have been identified. The most recurrent fusion partner in *MLL* rearrangements (*MLL*-r) is *AF4*, mapping at chromosome 4q21, accounting for approximately 36% of *MLL*-r leukemia and particularly prevalent in *MLL*-r acute lymphoblastic leukemia (ALL) cases (57%). *MLL*-r leukemia is associated with a sudden onset, aggressive progression, and notoriously poor prognosis in comparison to non-*MLL*-r leukemias. Despite modern chemotherapeutic interventions and the use of hematopoietic stem cell transplantations, infants, children, and adults with *MLL*-r leukemia generally have poor prognosis and response to these treatments. Based on the frequency of patients who relapse, do not achieve complete remission, or have brief event-free survival, there is a clear clinical need for a new effective therapy. In this review, we outline the current therapy options for *MLL*-r patients and the potential application of CAR-T therapy.

## 1. Rearrangements of the *MLL* Gene in Leukemia

Rearrangements of the *mixed-lineage leukemia* (also known as *MLL, KMT2A, HRX,* or *ALL1*) gene are found in *de novo* and therapy-related myeloid and lymphoblastic leukemias, accounting for 9% of adult cases, 3–5% of children [1,2,3,4] and 61–80% of infants in acute lymphoblastic leukemia (ALL) [5,6,7], as well as 5–11% of adult cases, ~15% of children [8,9,10,11] and 33–75% of infant cases in acute myeloid leukemia (AML) [12,13]. *MLL* rearrangements (*MLL*-r) are the most prevalent abnormalities in infants and represents one of the most aggressive leukemia subtypes, generally characterized by a rapid onset, hyperleukocytosis, and dismal prognosis [2,14]. Adult forms, often arising as secondary therapy-related leukemia, which is an aggressive form of AML following DNA topoisomerase II inhibitor treatments, are also associated with generally poor clinical outcomes [15,16]. At the cytogenetic level, the majority of *MLL-*leukemias are characterized by balanced chromosomal translocations involving the *MLL* locus mapping at 11q23 [3]. Owing to its designation of ‘promiscuous’ gene, *MLL* has been shown to rearrange with more than 80 distinct partner genes, of which the most frequently observed are *AF4*, *AF9*, *ELL,* and *ENL*, resulting in the translocations t(4;11)(q21;q23), t(9;11)(q22;q23), t(11;19)(q23;p13.1), and t(11;19)(q23;p13.3), respectively [17].

**Figure 1:**
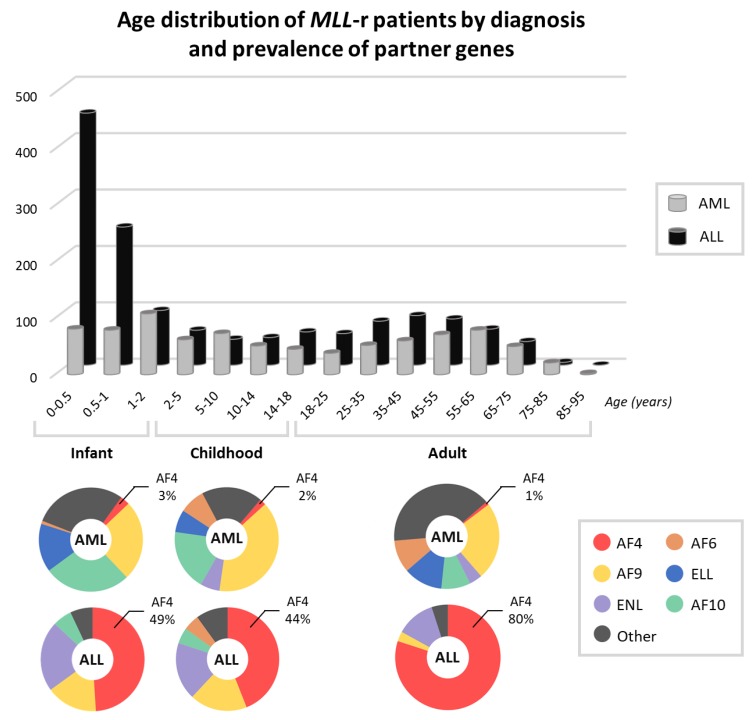
The age distribution of 2345 patients with *MLL* rearrangements (*MLL*-r) by diagnosis (acute myeloid leukemia (AML) or acute lymphoblastic leukemia (ALL)), as analyzed by Meyer et al. [17]. Above, the distribution and prevalence of the most frequent *MLL* partner genes are subdivided on the basis of age groups in ALL and AML. Age groups are defined as: infant (0–2 years old), pediatric (2–18 years old), and adult (>18 years). Overall, AF4 is the one most frequent gene in the majority of the subgroups, with the exception of AML in all age groups, in which AF9 and AF10 prevail. Figure adapted from [17].

The incidence and prevalence of individual subtypes and the corresponding diagnosis vary between adult and pediatric forms [18]. The distribution and prevalence of these subtypes are shown in Figure 1. Phenotypically, leukemic cells with *MLL*-r exhibit a heterogeneous lineage of pro-B or pro-B/monocytes. Certain phenotypes are more likely to be associated in conjunction with specific partner genes; for example, *AF4* is more prevalent in pro-B leukemias [19]. Switches between lineages, referred to as ‘lineage plasticity’, have been reported in patients and are a major therapeutic challenge [20,21]. The World Health Organization (WHO) classification recognizes *MLL*-leukemias as a recurrent genetic abnormality under AML, B-cell acute lymphoblastic leukemia (B-ALL), and acute leukemias of an ambiguous lineage [22].

The oncogenicity of translocations involving *MLL* is attributed to the generation of chimeric proteins via the in-frame fusion of the N-terminus of *MLL* with the C-terminus of the partner [23]. The current understanding of MLL-driven leukemogenesis points at a dysregulation in gene expression (e.g., *Hox* genes, among others) by the disruption of epigenetic mechanisms and chromatin status. Wild-type MLL is involved in transcriptional regulation and chromatin modifications for the establishment of cell-specific transcriptional programs (or ‘transcriptional memory system’), with a major role in embryogenesis and maintenance of embryonic and adult hematopoiesis. When disrupted due to a translocation, the crucial MLL regulatory domains (e.g., DNA binding, histone marking/recognition, transactivation) become disrupted and fused to a partner gene. Most MLL partners (i.e., AF4, AF9, ENL, ELL, and AF10) are also regulators of transcription by direct or indirect interaction with RNA polymerase II. The resulting MLL chimeras are capable of subverting crucial transcriptional machinery, altering global gene expression and epigenetic signatures of the affected cells. This ultimately results in strongly enhanced and improper expression of genes involved in proliferation and lineage identity, conferring stem cell-like properties and consequent transformation [24,25,26].

## 2. Leukemia with t(4;11)(q21;q23): Clinical Picture and Risk Stratification

**Figure 2:**
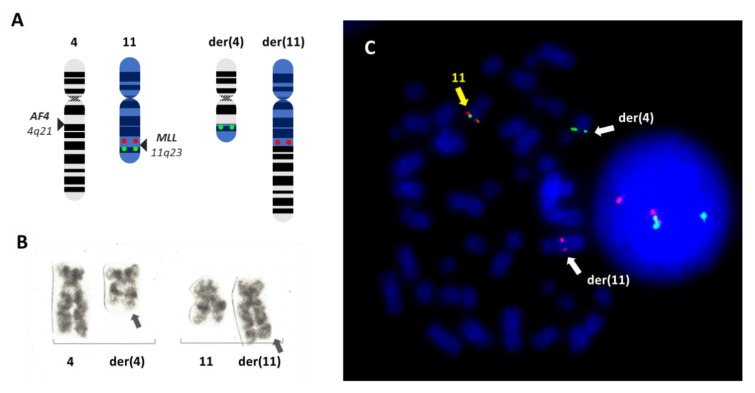
The t(4;11)(q21;q23) rearrangement involving *MLL*. Schematic representation of the translocation between chromosomes 4 and 11 giving rise to the two derivative chromosomes der(4) and der(11) (**A**). The location of the fluorescence *in situ* hybridization (FISH) probe XL MLL (Metasystems) is also indicated on the normal chromosome 11, consisting of one green and one red signal flanking the *MLL* locus at 11q23. In the event of the translocation, the two signals split, indicating the disruption of the *MLL* locus. As a result, the der(11) retains the red signal proximal to *MLL*, while the der(4) will contain the green signal corresponding to the distal portion of *MLL*. These patterns are visible in a representative metaphase from the RS4;11 cell line, known to harbor a t(4;11)(q23;q21), hybridized with the XL MLL probe (**B**). The same rearrangement is shown in (**C**), obtained by G-banding.

The t(4;11)(q21;q23) (Figure 2) represents one of the most recurrent translocations involving *MLL* and is most prevalent in lymphoblastic leukemia in both adults and infants/children. Clinically, the phenotype of patients with t(4;11) is B-ALL, with rare cases of AML [14,27]. As with other *MLL* rearrangements, t(4;11)-positive blasts present as mixed-lineage, morphologically lymphoblastic but exhibiting lymphoid and myeloid markers on the cell surface, such as CD19^+^/CD10^−^ and CD15^+^ and CD33^+^, respectively [14,28,29]. The translocation produces the MLL-AF4 chimeric protein by the fusion of the two loci at 11q23 and 4q21 on the derivative chromosome 11 [17,30]. While the production of the reciprocal AF4-MLL from derivative 4 is also possible, *AF4-MLL* transcripts are rarely found, as the fusion does not occur in-frame in all cases [31]. The chimera MLL-AF4 is considered to be a major contributor in initiating and maintaining the malignancy, although it is not capable of initiating the malignancy *per se* [32]. The mutational landscape of *MLL-*leukemias is extraordinarily simple, with a surprisingly low frequency of secondary mutations that may contribute to leukemogenesis [33,34]. For this reason, the mechanism by which the t(4;11)(q21;q23) abnormality promotes such an aggressive and clinically challenging phenotype remains unclear [35]. The role of the reciprocal fusion *AF4-MLL* is also a topic of debate [36,37,38,39].

Patients with t(4;11) generally present at diagnosis with a high WBC count (median 180,000/µL), FAB subtype L1 or L2 of B-cell lineage, and an immature B cell immunophenotype with frequent co-expression of myeloid markers (CD10^−^, CD19^+^, HLA-DR^+^, CD15^+^, CDw65^+^). Central nervous system (CNS) involvement and hepato-, spleno-, and lymphadenomegaly are often described [14], with CNS infiltration being notably common in children with t(4;11) [6]. As introduced before, rearrangements of the *MLL* gene and particularly the t(4;11) are notoriously linked to poor prognosis in both pediatric and adult forms, although differences exist between age groups. In the context of t(4;11), the poorest clinical outcomes are reported in infants below the age of 1 and adults >25–30 [40,41,42]. Conversely, children 1 to 9 years old exhibit better recovery rates [43,44]. From a biological point of view, gene expression analyses suggest that the development of MLL-driven leukemia in infants is distinct from older children, which could explain the marked, age-dependent differences observed in clinical outcomes [45]. In *MLL*-r infants, age <6 months, an extremely high WBC count (≥200,000–300,000/μL), lack of expression of CD10, and poor response to induction therapy are especially indicative of dismal prognosis [5,42,46,47,48,49], while in older patients with t(4;11) only in ages above 25 years old appear to be a significant predictor of poor outcome [41]. Nonetheless, the overall low frequency of adult *MLL*-r patients has hindered the identification of risk markers of high statistical significance, although older age is an established high-risk factor in adult ALL [50,51]. While rare, t(4;11) AML cases are equally associated with dismal prognosis [52] and deemed high risk according to the standard unfavorable prognostic factors for AML, namely ages above 60 years old, high WBC, and therapy-related AML [46].

## 3. Current Therapy for *MLL*-r Acute Leukemia Patients

Currently, treatment options for acute leukemias (AML or ALL) are not optimized for patients harboring *MLL* rearrangements. The *MLL*-r subgroup is cytogenetically heterogeneous and still hard to treat, with poor treatment outcomes from the available therapies and very short overall survival (OS). Furthermore, no specific treatment options exist for cytogenetic subgroups, including the t(4;11) [41]. Under most protocols, all *MLL* rearrangements are classified as intermediate-risk cytogenetic abnormalities, except t(4;11), t(6;11), and t(10;11) being recognized as adverse risk groups [52].

### 3.1. Cytotoxic and Cytoreductive Chemotherapy

Despite great advances in the understanding of targetable biological mechanisms underlining certain leukemia subtypes (e.g., tyrosine kinase inhibitors against *BCR/ABL1* fusions and all trans retinoic acid for acute promyelocytic leukemia *PML/RARA*), the treatment strategy for most patients with acute leukemia still relies on cytotoxic and cytoreductive chemotherapeutic drugs [53,54]. More recently, while targeted agents against specific mutations (e.g., FLT3-ITD or IDH1/2 in AML, CAR-T therapy in ALL) have opened new therapeutic options for some patients, the majority of patients are still treated according to standard chemotherapeutic protocols.

For ALL (with the exclusion of Philadelphia chromosome-positive patients), the standard protocols include an induction with a steroid (dexamethasone or prednisolone) in combination with vincristine (V+P). Asparaginase and an anthracycline (daunorubicin or doxorubicin) are added according to risk groups. Following induction chemotherapy, all patients with ALL receive CNS prophylaxis, this is followed by post-induction consolidation and maintenance chemotherapy. Allogeneic stem cell transplant in first CR is recommended for all adults in all age categories as it has shown to improve survival, while it is not recommended for children with standard risk ALL in first complete remission (CR) (may be recommended for certain subgroups in their first CR such as those with the Philadelphia chromosome positive ALL), and recommended for children who relapse after achieving initial remission. For AML, aggressive induction chemotherapy consisting of cytarabine infusion plus an anthracycline (e.g., daunorubicin or idarubicin) is used for remission induction. Post remission therapy after achieving CR is controversial and depends on several factors such as age, general condition or performance status. Some patients with favorable-risk AML and those who are not proceeding to hematopoietic stem cell transplantation (HSCT), receive intensive consolidation with high dose cytarabine following induction therapy. Patients who are eligible for HSCT proceed to transplantation following their induction therapy. The chemotherapy regimens of choice correlate with the wider risk stratification groups but are not specific to individual cytogenetic entities [55]. Furthermore, although protocols are adjusted by age and body mass, no age-specific drugs are available despite that the biology of infant leukemia is known to be genetically distinct from other age groups, and particularly for *MLL*-r leukemias [45].

In *MLL*-r patients, treatment with induction combination chemotherapy according to risk stratification achieves up to 90% of patients in total remission but are followed with high rates of relapse—a trend that was reported in several studies. Five-year event-free survival (EFS) in *MLL*-r infants is considerably lower than non-*MLL*-r patients. Although aggressive induction achieves excellent rates of CR (80–90%), *MLL*-r infant patients are prone to relapse, failure of second remission, and low overall survival [6,56]. A similar picture is seen in adult *MLL*-r patients, as initial CR in the majority of patients follows high rates of relapse and 30% OS, with the t(4;11) subgroup being the highest risk group for disease reoccurrence [41,57]. The tendency to relapse in these patients is indicative of chemoresistant leukemic cell populations that fail to be eradicated by chemotherapeutic agents, rendering this therapeutic approach unsuitable for achieving a durable cure [58,59]. Interestingly, some *MLL-*r ALL patients respond more positively to AML-oriented or AML/ALL-hybrid chemotherapeutic regiments, which is attributable to the biphenotypic and early progenitor properties of *MLL*-r leukemias [6,56], as demonstrated by the sensitivity of *MLL*-r ALL blast to cytarabine *in vitro* [60]. A meticulous determination of prevalence of lymphoid versus myeloid blasts in individual patients could dictate the most optimal AML/ALL hybrid protocols to follow [61], although no benefit of hybrid and AML-oriented protocols was shown in the Interfant-06 study [62]. It has also been shown that pediatric protocols may be more effective in young adults up to the age of 25 [63,64,65].

Toxicity and long-term complications are an obvious concern in the use of cytotoxic and cytoreductive chemotherapy, which have led to prospective investigations on minimal effective dosages and number of cycles [6,47,66,67]. The risk/benefit evaluation of more aggressive interventions are unclear, especially in the infant cohorts where therapy-related mortality has been reported as high as 10% [6,62,67], and a dose reduction in induction chemotherapy was correlated with similar CR outcomes and lower fatality [68].

### 3.2. Other Treatment Strategies

Despite the efforts in improvements and adaptation of regimens over the years, the use of chemotherapeutic agents has proven inefficient in achieving satisfactory cure rates for *MLL*-r patients [61,62]. Additional combinatory agents are being investigated to complement the cytotoxic activity of current protocols. Various attempts have been reported in selectively targeting the oncogenic MLL-AF4 fusion, as well as related proteins, pathways, and complexes, but these have not been successfully translated into clinical use yet (reviewed by Steinhilber and Marschalek, 2018) [26]. An attractive therapeutic target for *MLL*-r leukemia is the epigenetic dysregulation brought about by MLL fusions, which could be treated by the use of nucleoside analogs hypomethylating agents such as decitabine, azacytidine, and clofarabine (NCT02828358) [69,70]. Decitabine, in particular, was shown to exert anti-proliferative action in cell lines and in patient-derived xenografts bearing t(4;11) [71]. A wide range of potentially targetable epigenetic and chromatin regulators have been identified, many of which have not reached clinical stages as of yet [72,73]. An example that has undergone clinical trials and shown limited improvements for *MLL*-r are DOT1L (a histone methyltransferase) inhibitors [74,75]. Histone deacetylase (HDAC) inhibitors, such as vorinostat and romidepsin, are also of clinical interest, following promising *in vitro* studies demonstrating anti-leukemic activity in cell lines and patient samples with *MLL*-r including t(4;11) [76,77], as well as in vivo with the HDAC inhibitor panobinostat [78].

The extraordinarily simple mutational landscape of *MLL*-r leukemias poses an additional challenge in the identification of druggable targets [34]. Infant *MLL*-r ALL, in particular, holds the title for one of the lowest number of somatic mutations across all malignancies [34,79], highlighting the limited window of genetic and molecular therapeutic targets for these patients. Between 30–50% of *MLL*-r patients harbor N-RAS and K-RAS mutations, which, however, do not appear to be essential in leukemogenesis, as they are subclonal or lost at relapse [79,80,81]. In the case of t(4;11) patients, approximately 30–40% have RAS mutations [80,81]. The overexpression of the Fms-like receptor tyrosine kinase-3 (*FLT-3*) in *MLL*-r leukemia, which results in the constitutive activation of FLT-3 signaling, could provide an opportunity for therapeutic targeting [82]. It is, however, a topic of debate whether the overexpression of FLT-3 results from activating mutations, as some groups argue that FLT-3 mutations are uncommon [83] or even completely absent in *MLL*-r ALL [84,85,86]. The high expression of FLT-3 in t(4;11) patients has been suggested to derive from FLT-3 being a direct transcriptional target of MLL-AF4 [87], and they act cooperatively in impairing differentiation but not overproliferation and transformation [88]. Nevertheless, the FLT-3 inhibitor lestaurtinib did not benefit infants with ALL in a trial by the Children’s Oncology Group [89]. A phase I trial testing a new generation FLT-3 inhibitor quizartinib showed promising outcomes in children with *MLL*-r ALL or refractory AML [90], although concerns on resistance in AML have been reported [91].

### 3.3. Hematopoietic Stem Cell Transplant

Allogeneic and autologous hematopoietic stem cell transplant is one of the few curative treatments for acute leukemia and often the sole option for refractory/chemoresistant patients. Due to its classification in poor risk cytogenetics, patients with t(4;11) are recommended to receive HSCT as intensification treatment. Since a large proportion of *MLL*-r patients achieve first CR, the major clinical question has revolved around the benefit of HSCT versus chemotherapy as consolidation options to prevent relapse. However, conflicting evidence has been gathered on the efficacy of HSCT in the treatment of all *MLL*-r leukemias [43,92,93].

According to the Interfant-99 international ALL trial, the majority of infant *MLL*-r patients did not benefit from HSCT over standard consolidation chemotherapy [43], which was in agreement with other studies [42,67,94,95]. Nonetheless, only a sub-cohort of *MLL*-r infants with high-risk factors (<6 months of age, high WBC count, and/or poor response to steroidal induction) showed improved outcomes with HSCT in Interfant-99 [43], while Pui et al. (2002; 2003) [40,42] reported that infants with t(4;11) receiving HSCT had worse OS than those treated with chemotherapy only. Other studies concluded that HSCT is beneficial in improving survival in *MLL*-r infants at first CR [96] and following high intensity induction chemotherapy [97,98].

Limited studies have addressed the benefits of HSCT in adult *MLL*-r patients with ALL, with inconclusive results. An improvement in OS and three-year relapse in comparison to chemotherapy was only reported by Yu et al. (2014) [99] for *MLL*-r patients. In adult t(4;11) patients, not only did HSCT not outperform consolidation chemotherapy, but mortality following HSCT was reported to be 30% [41]. Patients under 25 years of age were, however, good responders to HSCT with a low rate of relapse (6%) [41]. In earlier studies, HSCT was shown to be beneficial for t(4;11) patients [100] or only *MLL*-r patients with further high-risk markers [101]. In adult AML patients with *MLL*-r, HSCT was shown to be beneficial following the first CR [102].

While the *MLL* status alone is generally indicative of poor risk, it is apparent that the risk stratification within the *MLL*-r group is not sufficiently defined to identify sub-risk factors that influence outcomes for HSCT. Even within the established high-risk cytogenetics, including t(4;11), the response to HSCT is heterogeneous and unpredictable based on current risk evaluators (i.e., WBC, age, initial treatment response). The feasibility of monitoring minimal residual disease (MRD) by quantification of the *MLL-AF4* fusion at the molecular level (e.g., by RT-PCR) has been shown to be a reliable evaluation marker in eligibility of HSCT in t(4;11) patients [103]. In most practices, all patients in high-risk groups are recommended for HSCT regardless of MRD, which may increase the risk of subjecting patients to unnecessary HSCT-related complications (e.g., graft-versus-host disease). HSCT and the preparatory procedures, which include radiation and chemotherapy, are also attributable to toxicity and late-onset adverse effects, which are of special concern in pediatric patients [92,104].

The success of HSCT is also attributable to the ’graft-vs-leukemia’ effect, by which the donor marrow cells exert anti-leukemic activity against leukemic cells in the recipient; in other words, an opposite mechanism to the graft-vs-host complication of transplants [105,106]. The culprit behind the variable response of *MLL*-r patients to HSCT could be linked to a resistance mechanism of these leukemias to the benefits of graft-vs-leukemia phenomenon. Tamai et al. (2010) [107], for instance, described a potential mechanism of apoptotic escape in cell lines with t(4;11) via tumor necrosis factor-alpha (TNF-α)-mediated pathways, which is known to be involved in graft-vs-leukemia cytotoxicity.

### 3.4. Immunotherapy

Immunotherapy including chimeric antigen receptor T cell (CAR-T) therapy for B-ALL is a new exciting approach. Although *MLL*-r patients have been permitted the use of this therapy, studies using CAR-T cell therapy have excluded infants <1 year with *MLL*-r, partially due to challenges in the production process when using autologous T-cells isolated from infants [61,108].

## 4. What Is CAR-T?

**Figure 3:**
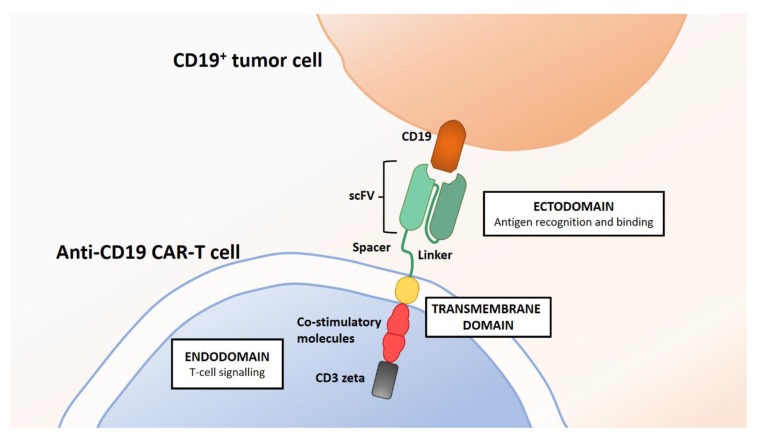
Interaction between anti-CD19 CAR-T cell receptor and CD19 antigen-presenting tumor cell. Chimeric antigen receptors on the anti-CD19 CAR-T cells consist of extracellular, transmembrane, and endodomain/intracytoplasmic regions, which are inserted into T cells to form an antigen recognition domain for a chosen target. This antigen recognition site is connected to the intracellular components, which triggers the activation of the T cell when in contact with the target antigen to destroy the tumor cell via cytokine killing. The single chain variable fragment (scFv) in a CAR is a chimeric protein consisting of the variable domains of the light and heavy chains of an antibody/immunoglobulin bound by a linker peptide. Its function is to recognize and bind to the chosen tumor-specific antigen. The spacer provides the bridge between the scFv and the transmembrane domain [109,110,111,112].

Chimeric antigen receptor T cell (CAR-T) therapy is an immunotherapy whereby the body’s own immune system is harnessed to target and destroy cancer cells [109]. Previous immunotherapies have relied on the use of monoclonal antibodies against tumor-associated targets, most notably CD19. The principle behind CAR-T, however, involves autologous CD4^+^ and CD8^+^ T lymphocytes being engineered with receptors specific to an antigen present uniquely on cancer cells, enabling the recognition of the particular antigen to selectively target the malignant cells. Chimeric antigen receptors (CARs) are synthetically engineered receptors that are grafted to T lymphocytes creating specificity to the chosen target antigen on cancer cells. The CARs consist of three components: an ectodomain, transmembrane domain, and endodomain, as illustrated in Figure 3.

The endodomain components have varied throughout the development of CARs in order to produce a more effective response. The most recent generation of CAR (fourth) consists of a co-stimulatory molecule such as CD28 and an interleukin-12 (IL-12) that connects to the CD3 zeta immunoreceptor tyrosine-based motifs [111]. The fourth generation of CARs are referred to as T cells redirected for universal cytokine-mediated killing (TRUCKs) because of their ability to increase T cell activation and trigger the destruction of antigen-presenting cancer cells by native immune cells [111].

**Figure 4:**
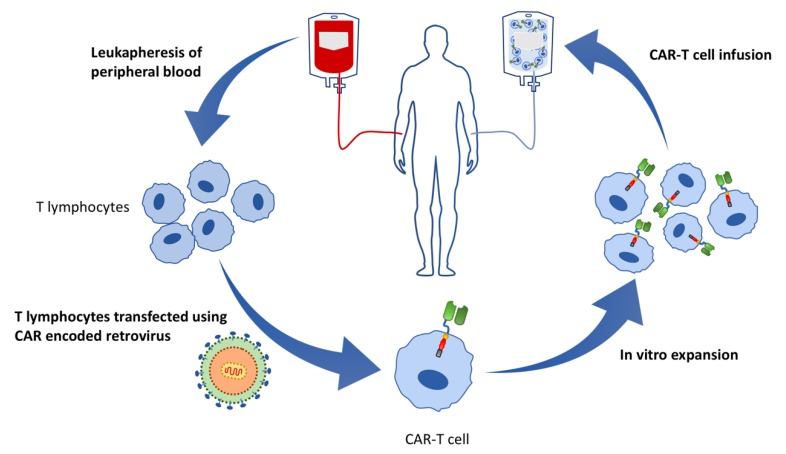
Cycle of the procedure of CAR-T cell production and infusion. Commencing with the harvesting of peripheral blood, from which T cells are separated by leukapheresis. The T lymphocytes are transfected by a viral or non-viral vector, e.g., retrovirus, carrying the genes encoding the CAR sequence in order to modify the T cells with the CAR component. The CAR-T cells undergo expansion and are then infused into the patient.

The process from CAR-T cell production to administration is a multifaceted procedure, as displayed in Figure 4. Firstly, leukocytes are removed from the patients’ (autologous) or donors’ (allogeneic) peripheral blood by leukapheresis/apheresis. The CD4^+^ and CD8^+^ T lymphocytes are then separated from the leukocytes and are transfected with a CAR encoded viral or non-viral vector. The modified T cells (CAR-T cells) undergo ex vivo proliferation and purification until the required quantity is produced [103,105]. Following the ex vivo engineering procedure, the modified T cells are subsequently reinfused back into the patient [109,111].

The selection of appropriate tumor-associated antigens is of utmost importance in ensuring a targeted activity against malignant cells only. Currently, CD19 is used as the target antigen in B-ALL; however, other extracellular receptors are also being investigated to expand the use of CAR-T therapies against tumor cells not expressing CD19 [109,113]. CD19 is used as a target antigen because of its lineage selectivity and relatively universal expression on the cell surface of B cells and absence from hematopoietic stem/progenitor cells and non-hematopoietic tissues [114]; therefore, the CAR-T cells are able to target only cancer cells in B-ALL, minimizing the destruction of healthy cells [112].

In August 2017, the FDA approved tisagenlecleucel (Kymriah), the first CAR-T cell therapy for the treatment of relapsed and refractory B-ALL [115]. Tisangenlecleucel is currently indicated for CD19^+^ B-cell ALL patients who are under 25 years old and have relapsed twice or more or relapsed following an HSCT. Tisagenlecleucel has also received approval from the European Commission and has the recommendation of marketing authorization by the European Medicines Agency. Studies conducted in ALL patients who previously have been heavily pre-treated have shown a very high CR rate reaching 91% [116], with some studies showing remissions lasting up to 2 years [117]. The only other clinically approved CAR-T therapy is axicabtagene-ciloleucel (Yescarta) for the treatment of selected CD19^+^ B-cell lymphoma subtypes in adults [118].

## 5. Treatment of *MLL*-r with CAR-T Cell Therapy: Advantages and Challenges

CAR-T has shown great potential as seen with the impressive rates of CR achieved in patients who have relapsed following other treatments, with the potential of fulfilling the clinical gap in the treatment of refractory t(4;11) patients. CAR-T possesses several favorable attributes compared to the standard protocols, including the ability to treat patients irrespective of their human leukocyte antigen (HLA) haplotype through a major histocompatibility complex-independent antigen recognition method, unlike other immunotherapies [119]. As a result of the therapy’s ability to target a specific antigen with single-cell lineage or universal tumor specificity, the toxicity and lethality against healthy cells are lessened compared to chemotherapeutic agents. For *MLL*-r patients, whose cytogenetic and phenotypic manifestations are variable, treatment with CAR-T offers the opportunity for personalized medicine by identifying the key target for each patient through immunophenotypic and cytogenetic techniques. However, despite these promising findings in recent clinical studies, there are some challenges that must be addressed. A summary of the advantages and challenges is outlined in Table 1.

The proportion of *MLL*-r patient enrolled in CAR-T studies is limited. In a study conducted at the Memorial Sloan Kettering Cancer Center, out of 16 patients, one had *MLL*-r in the form of t(9;11), and another patient had an *MLL* deletion. The overall CR for all patients was 85%, with the six-months survival being 58%. The patient with *MLL*-r t(9;11) achieved MRD; however, the patient with the *MLL* deletion showed no response to the treatment [120]. Gardner et al. [121] reported the outcome of the treatment of seven B-ALL patients harboring an *MLL*-r with CD19-targeted CAR-T cells. All patients first underwent chemotherapy, followed by the infusion of the CD19-CAR-T cells. Following the treatment, all seven patients achieved CR in the blood and bone marrow. One month after treatment, two of the seven patients, one harboring t(4;11), developed AML. The AML clonally related to the original B-ALL, which was considered a unique mechanism of CD19 immune escape in *MLL*-r leukemias, as this event was not observed in any B-ALL patients without *MLL*-r in the study [121]. The age restriction of <25 applied to current clinically approved CAR-T therapies is a major drawback for the older adult t(4;11) cohort, who are poor responders to standard treatments and still in need of more effective interventions [41]. Also, the exclusion of infants below the age of one from the majority of CAR-T clinical trials is discouraging for the highest risk group of under six months. From a technical point of view, the process of manufacturing sufficient amounts of autologous T cells is often challenging, especially in heavily pre-treated patients and infants suffering from therapy-related lymphopenia. As a solution to these limitations, Qasim et al. [122] used gene editing to generate universal CAR19 (UCART19) allogeneic T cells from healthy donors to be administered as ‘off-the-shelf’ CAR-T cells. UCART19 were successful in achieving remission in two infants with relapsed refractory CD19^+^ B-ALL, in conjunction with chemotherapy and anti-CD52 serotherapy. UCART19 therapy for adult and pediatric relapsed/refractory B-ALL is currently under clinical investigation (NCT02735083, NCT02808442, NCT02746952).

### 5.1. Immune Escape

Loss of the CD19 antigen and immune escape is a crucial obstacle for *MLL*-r patients and especially the t(4;11) subtype. The escape of *MLL*-cells targeted by anti-CD19 immunotherapy is not unprecedented, as earlier reports have described similar issues with the anti-CD19-directed antibody blinatumomab by the loss of CD19 antigen expression [21,123]. Several mechanisms have been proposed for CD19 antigen loss in B-ALL following CD19-targeted therapies, including mutations or alternative splicing of CD19, antigenic masking, and lineage switch (reviewed by Shah et al.) [124]. As discussed earlier, the distinctive biphenotypic nature of *MLL*-r leukemias has been shown to confer the ability to switch between lineages in affected leukemic cells under selective pressure. This peculiar ability, which is seen inpatient [20,21] and in vitro [125], poses a challenge in therapies based on immune recognition of cellular markers in *MLL*-r patients. Lymphoid-to-myeloid, but extremely rarely myeloid-to-lymphoid, phenotypic transitions in *MLL*-r patients have been observed following chemotherapy and HSCT [20], and recently, following CD19-CAR-T administration [121,126]. The mechanism behind the development of clonally-related AML in the study by Gardner et al. [121] is not clear, but the investigators suggested that immunological pressure by CAR-T cells selectively advantaged a rare myeloid clone, or it could represent a de-differentiation from a lymphoid blast following the treatment [20,121]. In t(4;11), MLL-AF4 is held responsible for the dictation of predominantly lymphoid lineage identity, and a subsequent shift to myeloid lineage is thought to result from additional mutational events that override the lymphoid identity [127,128]. In fact, loss of CD19 alone is not sufficient to induce lineage switch, as demonstrated by Jacoby et al. [129] in vivo in a non-*MLL*-r B-ALL murine model. *MLL*-r patients’ susceptibility to the CD19^−^ lineage switch could also be attributed to the intrinsic increased lineage plasticity of the *MLL*-r leukemic cell of origin, as the translocation of t(4;11) occurs at an early non-committed progenitor stage [81].

Lineage switch at relapse (irrespective of CD19-therapies) has been associated with the presence of an occult, multipotent pre-leukemic clone that follows the myeloid lineage over lymphoid by the acquisition of new genetic aberrations, which have recently been linked to an epigenetic dysregulation affecting transcriptional programs responsible for lineage identity [130]. In the case of post-CD19-CAR-T therapy, it remains to be elucidated whether the observed CD19 immune escape is a unique mechanism in response to CAR-T specifically. For instance, this may be associated with specific immunological alterations in the bone marrow microenvironment that confer a new context and possibly clonal advantage to CD19^−^ populations, which is also supported by the observations that microenvironment clues are able to modulate lineage identity and oncogenicity in *MLL*-r leukemia [127]. An interesting possibility is that a common side effect of CAR-T (in detail in 5.2), cytokine-release syndrome (CRS), could lay the ground for myeloid lineage switch. Supported by *in vitro* evidence on t(4;11) cell lines, it has been proposed that exposure to high levels of cytokines, such as interleukin 6 (IL-6), is able to induce myeloid differentiation [131].

### 5.2. Target Specificity and Alternative Antigens

Although CAR-T cell therapy displays impressive specificity against tumor cells, the majority of target antigens may also be expressed on healthy tissue; this leads to the targeting of normal cells producing an increase in toxicity. This is referred to as ‘on-target/off-tumor recognition’ [132]. While this still remains a concern, off-target toxicity is reduced compared to other less targeted therapies. Even the elimination of tumor cells can cause complications such as tumor lysis syndrome [133]. In the case of a CD19-specific target, some non-pathogenic B cells are lysed, often resulting in B-cell aplasia. This is of particular concern as the targeting of plasma cells may result in compromised humoral immunity. Despite the universality of CD19 on B cells of various maturation stages, CD19 expression decreases with differentiation. Plasma cells have been shown to exist as both CD19^+^ and CD19^−^ subsets, with the latter prevailing in the bone marrow and orchestrating long-term humoral responses [134]. In a study by Bhoj et al., anti-CD19 CAR-T therapy was shown to reduce circulating antibody levels but maintaining pathogen-specific antibodies stable, suggesting that CD19^−^ plasma cells are functional in sustaining long-term immunity even under CD19^+^ B cell aplasia [135]. More long-term observations are, however, needed to establish prolonged effects on the immune system. Interestingly, Garfall et al. [136] reported successful use of CD19-directed CAR-T in a myeloma patient with an extremely low proportion of CD19-expressing myeloma cells, suggesting that CD19^−^ populations are also attacked. Subsequent analysis by super-resolution microscopy revealed that seemingly CD19^−^ cells did express CD19 on the surface below the resolution of flow cytometry and qPCR and were, therefore, successfully targeted by CAR-T. Despite this study proving an exceptional threshold of recognition by CAR-T cells at less than 100 CD19 molecules per cell [137], the efficacy of CAR-T activity has been shown to be reduced at low antigen densities [138], but the thresholds are variable [139]. Interestingly, although CD19 is a characteristic marker in *MLL*-r leukemia, it does not seem to be indispensable for the B-ALL malignant phenotype in t(4;11) leukemia, as demonstrated in xenograft repopulation experiments [28,140]. This concept may be applicable in the design of the next generation of CAR-T therapies in order to identify the most appropriate antigens beyond CD19 for the *MLL*-r group [113]. The co-expression of myeloid antigens could qualify t(4;11) patients (as well as other *MLL*-r groups) for future multi-target or AML-oriented CAR-T interventions (e.g., anti-CD33) [124,141], similarly to how chemotherapeutic protocols have been adapted to *MLL*-r patients with AML/ALL hybrid regimens.

In the attempt of refining target specificity and circumventing antigen escape mechanisms, alternative t(4;11) tumor-specific antigens are being explored. CAR-T against CD22 have been developed following the observations that relapsed B-ALL with CD19 antigen loss retain the B-cell antigen CD22. Comparable to CD19-targeted CAR-T, CD22-CAR-T has shown promising results in both adult and pediatric patients, including relapses from previous CD19-directed therapies [142,143]. Nonetheless, low CD22 density, which is notorious of *MLL*-r leukemia [144], could render CAR-T cells less effective against the target. Modulation of antigen density on tumor cells, as demonstrated by Ramakrishna et al. for upregulation of CD22 using bryostatin 1, could increase success rates. Multi-target CAR-T against CD19 and CD22 simultaneously has been shown to achieve remission in a B-ALL adult patient [145]. An alternative multi-target CAR-T option specifically for *MLL*-r B-ALL is to target CD19 and CD133 [146]. CD133 is highly specific for *MLL*-r leukemic cells and particularly for the t(4;11) [146,147]. Nonetheless, it will require further evaluations to establish the feasibility of CD133 as a target to minimize potential on-target/off-tumor effects, as later reports by Bueno et al. (2019) [148] showed that CD133 appears to be expressed on healthy hematopoietic progenitors. Another attractive target is neuron-glial antigen 2 (NG2), which is a selective marker of *MLL*-r leukemia [149]. Lopez-Millan et al. [150] demonstrated that NG2 targeting resulted in the mobilization of *MLL*-r cells from the bone marrow into circulation in vivo, proposing that anti-NG2 therapies can selectively render *MLL*-r blasts more vulnerable to co-administered antileukemic agents. Also highly specific to *MLL*-r [151], chondroitin sulfate proteoglycan 4 (CSPG4) is a potential candidate target for *MLL*-r patients, with promising *in vitro* observations of CSPG4-directed CAR-T selective lysing cells harboring t(11;19) [152]. Overall, with high-resolution technologies, as demonstrated by Nerreter et al. [137], it will be possible to redefine the current immunophenotypic profiling of *MLL*-r cells to identify unique markers that fall under the current detection limitations of widely used techniques.

### 5.3. Toxicity

Toxicity following CAR-T cell infusion has many potential contributory causes and can have a multifactorial nature, the most prevalent being the triggering of cytokine-release syndrome (CRS). Cytokine-release syndrome is an inflammatory response initiated by the overproduction of cytokines induced by the binding of CAR-T cell receptors to its target antigen causing a cascade of activation and release of excessive levels of potent cytokines [153], including interleukin-6 (IL-6), interferon-gamma (IFNγ), and tumor necrosis factor (TNF) [153,154]. Severe CRS can be life-threatening and can also lead to hemophagocytic lymphohistiocytosis, an inflammatory syndrome caused by hypercytokinemia [155], and macrophage activation syndrome, a severe disorder caused by the dysregulation of T cell and macrophage activation [156]. Anaphylaxis can also occur as a result of the scFv on the CAR, often originating from a murine monoclonal antibody, which can be recognized as foreign [132]. CRS-associated neurotoxicity has also been reported in the form of delirium and epileptic episodes, which were, however, deemed reversible and clinically manageable [120,133,157,158,159], but may pose additional risks to patients with t(4;11) who often present leukemia-related CNS involvement. The identification of susceptibility factors for the development of CRS will improve the management of these complications.

### 5.4. Persistence and Long-Term Effects

CAR-T cell persistence is still an area of debate as the duration of which CAR-T cells remain in the blood varies based on multiple factors, including the co-stimulatory signaling molecules selected for the endodomain of the CAR structure. Certain co-stimulatory domains appear to improve CAR-T cell persistence, such as 4-1BB (CD137) [160]; however, it is unclear whether this increased longevity facilitates sustained remissions. Inconsistencies between cases are often observed; a study by Maude et al. reported the average duration of persistence of CD19-specific CAR-T cells in the blood to be 168 days in the 60 patients tested [161]. In contrast, a study by Davila et al. showed that the CAR-T cell levels were low to undetectable after 2–3 months [120]. This emphasizes the need for the proper CAR structure/design to be chosen for the best outcome. Despite the differences in persistence, a long-term follow-up study found no association between CAR-T cell persistence and longevity of remission [162].

## 6. Conclusions

In conclusion, CAR-T therapy is currently the most effective therapy for patients with CD19-positive B-ALL who have failed previous lines of therapy. The majority of the studies did not pro-actively select patients with *MLL*-r, which affected the number of cases harboring this rearrangement enrolled and, consequently, our ability to fully assess the benefit/risk of this treatment for patients with t(4;11). Despite the small number of patients, CAR-T therapy has shown encouraging CR rates. Unfortunately, *MLL*-r patients are prone to the unique immune escape from CD19-targeted immunotherapies by clonal evolution and lineage switch. The benefit/risk assessment of the treatment of *MLL*-r acute leukemias with CAR-T cell therapy can only be fully assessed with more studies with a sufficient number of patients with these rearrangements, and by identifying possible differences among cytogenetic subgroups. Long-term EFS and OS results still remain relatively undetermined on account of the novelty of this treatment. With particular focus on the cytogenetic subgroup t(4;11), the expression of CD19 allows the successful use of the current clinically approved CAR-T therapy, but the discovery of targetable antigens other than CD19 will expand its use in the clinic. The variability in clinical phenotypes associated with different *MLL* rearrangements is an obstacle in the accessibility of CAR-T for all *MLL-*r patients. Hence, more studies involving *MLL*-r patients will enable the identification of more efficient therapies using CAR-T or other targeted agents for this hard-to-treat subgroup of acute leukemias.

## Figures and Tables

**Table 1:** Advantages and disadvantages of the application of CAR-T cell therapy in the treatment of B-cell acute lymphoblastic leukemia (B-ALL).

Advantages	Disadvantages/Challenges
Independent of HLA and major histocompatibility complex	Toxicity/Neurotoxicity: ○ Cytokine-release syndrome ○ Macrophage activation syndrome/Hemophagocytic lymphohistiocytosis ○ Anaphylaxis
Avoid majority of unnecessary killing of healthy tissue	Lysis of some healthy cells expressing the target antigen
Numerous accounts of efficacy	Antigen escape/Lineage switch
Personalized treatment (many potential target antigens)	CAR-T cell persistence
Treatment of advanced ALL when there are no other effective available options	Cost of treatment is very high in addition to the need for hospitalization of the patients and close observation during and after treatment
	Current regulatory approved age restriction of <25 and >1 year old limits accessibility to more patients
